# Epithelial membrane protein 1 promotes tumor metastasis by enhancing cell migration via copine-III and Rac1

**DOI:** 10.1038/s41388-018-0286-0

**Published:** 2018-06-04

**Authors:** Mohammad Khusni B. Ahmat Amin, Akio Shimizu, Dimitar P. Zankov, Akira Sato, Souichi Kurita, Masami Ito, Toshinaga Maeda, Tetsuya Yoshida, Tomohisa Sakaue, Shigeki Higashiyama, Akihiro Kawauchi, Hisakazu Ogita

**Affiliations:** 1 Division of Molecular Medical Biochemistry, Department of Biochemistry and Molecular Biology, Shiga University of Medical Science, Otsu, Japan; 2 Department of Urology, Shiga University of Medical Science, Otsu, Japan; 3 Division of Cell Growth and Tumor Regulation, Proteo-Science Center (PROS), Ehime University, Toon, Japan; 4 Department of Cardiovascular and Thoracic Surgery, Ehime University Graduate School of Medicine, Toon, Japan; 5 Department of Biochemistry and Molecular Genetics, Ehime University Graduate School of Medicine, Toon, Japan

## Abstract

Tumor metastasis is the most common cause of cancer death. Elucidation of the mechanism of tumor metastasis is therefore important in the development of novel, effective anti-cancer therapies to reduce cancer mortality. Interaction between cancer cells and surrounding stromal cells in the tumor microenvironment is a key factor in tumor metastasis. Using a co-culture assay system with human prostate cancer LNCaP cells and primary human prostate stromal cells, we identified epithelial membrane protein 1 (EMP1) as a gene with elevated expression in the cancer cells. The orthotopic injection of LNCaP cells overexpressing EMP1 (EMP1-LNCaP cells) into the prostate of nude mice induced lymph node and lung metastases, while that of control LNCaP cells did not. EMP1-LNCaP cells had higher cell motility and Rac1 activity than control LNCaP cells. These results were also observed in other lines of cancer cells. We newly identified copine-III as an intracellular binding partner of EMP1. Knockdown of copine-III attenuated the increased cell motility and Rac1 activity in EMP1-LNCaP cells. Reduced cell motility and Rac1 activity following knockdown of copine-III in EMP1-LNCaP cells were recovered by re-expression of wild-type copine-III, but not of a copine-III mutant incapable of interacting with EMP1, suggesting the importance of the EMP1–copine-III interaction. Phosphorylated and activated Src and a Rac guanine nucleotide exchange factor Vav2 were found to be involved in the EMP1-induced enhancement of cell motility and Rac1 activation. Moreover, EMP1 was highly expressed in prostate cancer samples obtained from patients with higher Gleason score. These results demonstrate that upregulation of EMP1 significantly increases cancer cell migration that leads to tumor metastasis, suggesting that EMP1 may play an essential role as a positive regulator of tumor metastasis.

## Introduction

Tumor metastasis is frequently observed in the course of malignant cancer progression and is the major life-threatening event in individuals with cancer [1, 2]. In the initial stages of tumor metastasis, cancer cells escape from the originating tumor site and invade into the surrounding tissues in which stromal cells exist. The physical and functional contact between escaped cancer cells and stromal cells contributes to the formation and enlargement of the tumor microenvironment, leading to alterations in the characteristics of cancer cells. It has recently been considered that the tumor microenvironment is an important biological concept behind the mechanism of cancer progression including tumor growth, spread, and metastasis [3–5]. Clinical and experimental studies have provided evidence that chemical communication between cancer cells and the surrounding microenvironment through growth factors and chemokines contributes to the regulation of cancer progression [6, 7]. However, little is known concerning the effect of physical contact between cancer cells and stromal cells on cancer progression, especially on tumor metastasis.

To address these issues, we developed an in vitro co-culture system using prostate cancer cells and prostate stromal cells, and examined the effect of direct physical interaction between these cells on their genome-wide gene expression profiles using DNA microarray assays. We focused on cell surface proteins in these assays, because they are reported to be involved in the regulation of cancer progression, including tumor metastasis, and represent a target of anti-cancer therapy through their approachable localization [8–10]. We found in this study that the expression of epithelial membrane protein 1 (EMP1) was highly induced in cancer cells, and subsequently investigated its role in tumor metastasis.

## Results

### Identification of genes with upregulated expression following contact between cancer cells and stromal cells

**Fig. 1:**
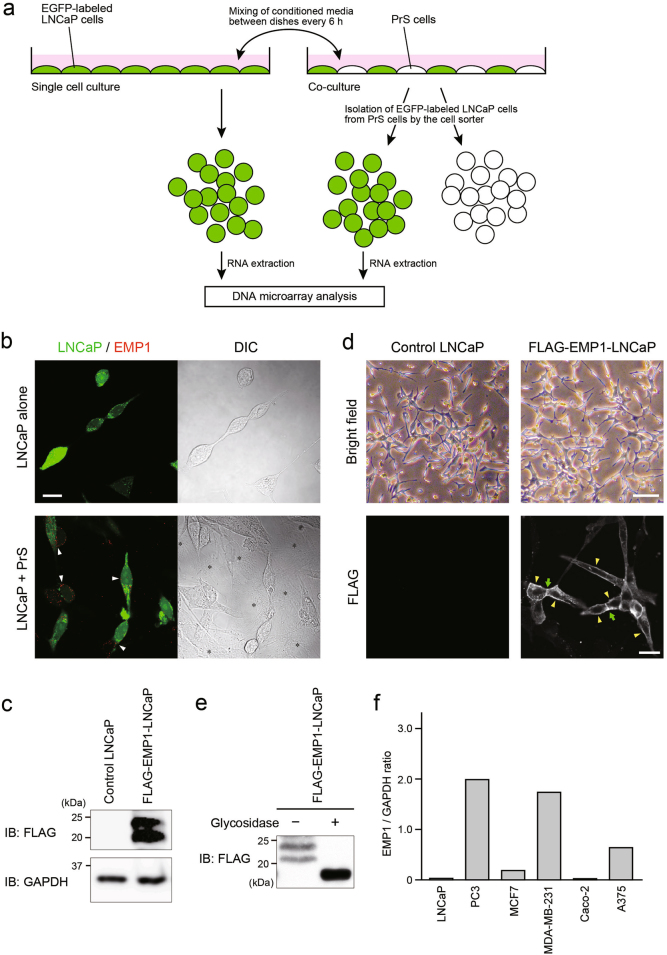
Identification, expression, and localization of EMP1. **a** Schematic illustration of the co-culture assay used to identify genes with upregulated expression following the interaction of cancer cells (LNCaP cells) with stroma cells (PrS cells). **b** Cell morphology and EMP1 expression in LNCaP cells co-cultured with or without PrS cells were observed by immunofluorescence microscopy. Arrowheads: EMP1 expression at the surface of LNCaP cells. Asterisk: PrS cells. DIC: differential interference contrast. Scale bar, 10 μm. **c** FLAG-EMP1 protein levels in LNCaP cells stably expressing FLAG-EMP1 (FLAG-EMP1-LNCaP cells) were determined by western blotting. **d** Cell morphology of control and FLAG-EMP1-overexpressing LNCaP cells was examined by light microscopy (bright field). Localization of FLAG-EMP1 in LNCaP cells was determined by confocal microscopy. Arrowheads and arrows indicate the localization of FLAG-EMP1 at the free cell surface and at the cell–cell junction site, respectively. Scale bars, 50 μm and 20 μm in the bright field and immunofluorescence images, respectively. **e** Lysates from FLAG-EMP1-LNCaP cells were treated with or without glycosidase, followed by western blotting. **f** Expression levels of EMP1 in several cell lines were analyzed by semi-quantitative PCR and normalized to *GAPDH* expression

We first sought to determine whether direct physical interaction between cancer cells and stroma cells could change the gene expression that affects metastatic potential of cancer cells. For this determination, we performed a co-culture assay using human prostate cancer LNCaP cells stably expressing enhanced green fluorescent protein (EGFP) and primary human prostate stromal (PrS) cells. These PrS cells were also used in the previous study in which PrS cells and prostate cancer cells were co-cultured [11]. As a control, EGFP-LNCaP cells were cultured alone. To exclude the effect of co-culture-mediated secretion of soluble factors such as cytokines and growth factors on cancer cell characteristics, conditioned media were regularly (every 6 h) mixed between the dishes of co-cultured cells and control cells (Fig. 1a). At 48 h after cell culture, co-cultured EGFP-LNCaP cells were isolated from PrS cells using a cell sorter. The gene expression profile of co-cultured LNCaP cells was compared with that of LNCaP cells cultured alone by the DNA microarray assay, and 30 genes were found to be upregulated more than threefold in co-cultured LNCaP cells (Supplementary Table S1). Among these genes, we selected a cell surface protein EMP1, which contains four transmembrane domains [12], for further examination, because little is known about the role of this molecule in cancer progression and metastasis.

### Characteristics of EMP1 for cancer progression

First, we confirmed that the protein expression of EMP1 was upregulated in LNCaP cells co-cultured with PrS cells (Fig. 1b). Cell morphology of LNCaP cells did not significantly change in the presence or absence of PrS cells. Then, to explore the characteristics of EMP1 in prostate cancer cells, we generated LNCaP cells stably expressing FLAG-tagged EMP1 (Fig. 1c), and observed the localization of FLAG-EMP1 at the plasma membrane (Fig. 1d). Because the doublet of the band for FLAG-EMP1 in western blotting disappeared in the presence of glycosidase, FLAG-EMP1 was considered to be glycosylated (Fig. 1e). Semi-quantitative PCR analysis revealed that endogenous expression of EMP1 was very low in LNCaP cells compared with other types of cancer cells (Fig. 1f). The PCR results also showed that the expression of EMP1 was strongly increased in highly malignant and metastatic cancer cells, such as PC3 and MDA-MB-231 cells, suggesting a pro-metastatic function of EMP1.

**Fig. 2:**
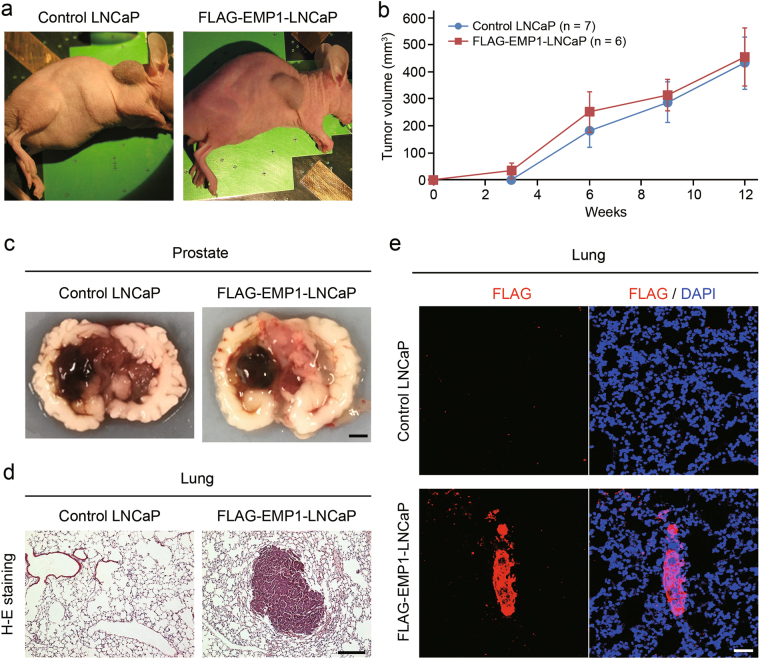
EMP1-mediated tumor growth and metastasis in nude mice. **a** Representative examples of tumor formation 12 weeks after subcutaneous injection of control or FLAG-EMP1-overexpressing LNCaP cells in nude mice. **b** Summary graph of tumor growth after injection of each type of LNCaP cells. **c** Tumor formation in the prostate of male nude mice 6 weeks after orthotopic injection of LNCaP cells with or without FLAG-EMP1 expression. **d** Detection of lung metastasis by H–E staining in nude mice injected with FLAG-EMP1-LNCaP cells. **e** Immunohistochemistry of the lung. Positive staining was observed in nude mice injected with FLAG-EMP1-LNCaP cells. Scale bars, 2 mm in **c**, 200 μm in **d**, and 50 μm in **e**

**Fig. 3:**
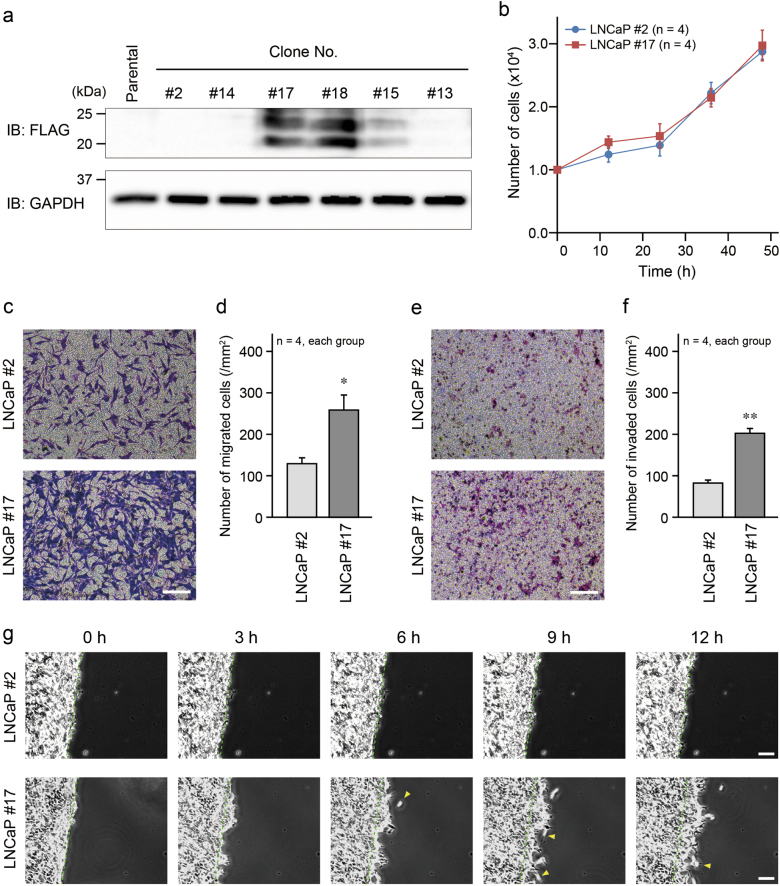
EMP1 induced increase in cell migration and invasion in LNCaP prostate cancer cells. **a** Several LNCaP clones stably expressing FLAG-EMP1 were verified by western blotting. **b** Proliferation of LNCaP cells with very low FLAG-EMP1 expression (clone #2) and high expression (clone #17) was assessed by cell counting at the indicated time points. **c, e** Representative results of the Boyden chamber and invasion assays. Cells migrated to the lower chamber were visualized by modified Giemsa staining. **d, f** Summary graph of migrated LNCaP cells in the Boyden chamber and invasion assays. *p < 0.05 and **p < 0.01 vs. LNCaP #2. **g** Collective cell migration assay. Confluent cell layers of each LNCaP cell clone were scratched, and cell migration was observed for 12 h by time-lapse light microscopy. Dotted green lines indicate the cell front immediately after scratching. Arrowheads indicate cells distant from the cell clump through particularly higher cell migration. Scale bars, 200 μm in **c** and **e**, and 100 μm in **g**

We next evaluated the tumor-forming ability of EMP1 in vivo. FLAG-EMP1-LNCaP cells or control LNCaP cells were subcutaneously injected into nude mice, and tumor growth was monitored for 12 weeks. The growth of tumors derived from FLAG-EMP1-LNCaP cells was similar to that from control LNCaP cells (Fig. 2a, b), suggesting that EMP1 had no effect on cell proliferation. This observation was also confirmed in vitro (Fig. 3b). Then, we investigated the metastatic property of EMP1 by orthotopic injection of LNCaP cells directly into the anterior prostate gland. This type of injection has been reported as the most effective route for examining prostate cancer progression [13, 14]. At 6 weeks after injection, we observed the formation of solid tumors at the primary site of injection of both control LNCaP cells (in 6 out of 13 mice) and FLAG-EMP1-LNCaP cells (in 11 out of 22 mice) (Table 1; Fig. 2c). The size of the tumors was similar between the two groups (Supplementary Table S2). No metastasis was detected in the lymph nodes or lung of the control group, while six mice injected with FLAG-EMP1-LNCaP cells exhibited metastatic lesions in the lymph nodes and/or lung; five mice in the lymph nodes, three mice in the lung, and two mice in both lymph nodes and lung (Table 1; Fig. 2d, e), suggesting that EMP1 could promote tumor metastasis.

**Table 1:** Metastatic activity of EMP1-overexpressing LNCaP cells

Cell type	No. of mice				
	Injected	Tumors in the prostate	Metastasis		
			Lymph node and/or lung	Lymph node	Lung
Control LNCaP	13	6	0	0	0
FLAG-EMP1-LNCaP	22	11	6*	5^a^	3^a^

^a^ Two mice have metastatic lesions in both lymph nodes and lung

* *p* < 0.05 vs. control LNCaP

### Mechanism of EMP1-promoted tumor metastasis

To further examine the mechanistic role of EMP1 in tumor metastasis, we generated clones of FLAG-EMP1-LNCaP cells and selected two of these clones: LNCaP #2 cells, which faintly expressed FLAG-EMP1, and LNCaP #17 cells, which expressed high levels of FLAG-EMP1 (Fig. 3a). Because these cells proliferated similarly (Fig. 3b), we tested their capacity for cell migration, a key factor in the regulation of metastasis [15, 16]. In the Boyden chamber assay, the number of LNCaP #17 cells that migrated to the lower chamber was significantly greater than that of LNCaP #2 cells (Fig. 3c, d). Similar to the Boyden chamber assay, the number of invaded cells was higher in LNCaP #17 cells than LNCaP #2 cells (Fig. 3e, f). The result of the collective cell migration assay was also consistent with the above experiments. Although LNCaP #2 cells hardly moved in this assay, LNCaP #17 cells migrated faster, and some of the cells migrated relatively far from the cell front (Fig. 3g; Supplementary Movies S1 and S2). These results indicated that overexpression of EMP1 may enhance the migratory response of cancer cells to metastasis.

### Copine-III as an intracellular binding partner of EMP1 for the transmission of EMP1-mediated signaling

**Fig. 4:**
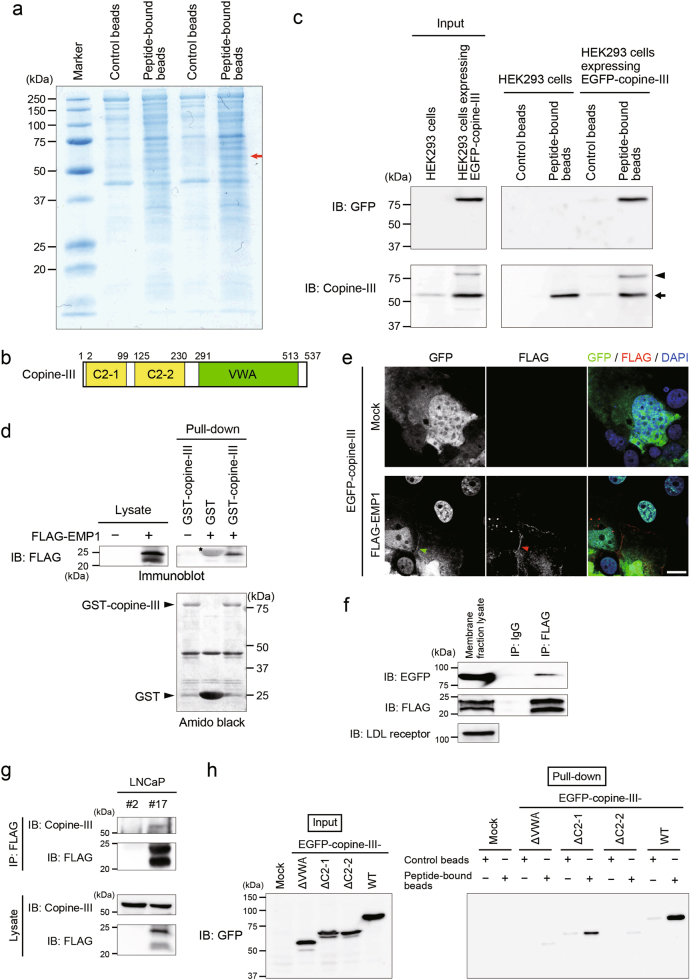
Identification of copine-III as an EMP1 interacting molecule. **a** Coomassie Brilliant Blue staining of LNCaP cell proteins pulled down with control beads or beads bound to polypeptides of the EMP1 intracellular region (peptide-bound beads). Arrow indicates copine-III, which was determined by mass spectrometry. **b** Schematic illustration of each domain of copine-III. Amino acid numbers indicate the beginning and end of each domain based on the UniProt database. C2-1: protein kinase C conserved region 2 domain-1, C2-2: protein kinase C conserved region 2 domain-2, VWA: von Willebrand factor type A domain. **c** Association of copine-III with the EMP1 intracellular region. The EGFP-copine-III plasmid was transfected into HEK293 cells, and the cell lysates were pulled down with the control or peptide-bound beads used in **a**, followed by western blotting. Arrowhead: ectopically expressed EGFP-copine-III; arrow: endogenous copine-III in HEK293 cells. **d** Association of copine-III with FLAG-EMP1. The FLAG-EMP1 plasmid was transfected into HEK293 cells, and the cell lysates were pulled down with GST-copine-III or GST as a control, followed by western blotting. Amido black staining was applied to detect the amount of GST-copine-III or GST used in the assay. Star: GST. **e** Localization of copine-III with EMP1. EGFP-copine-III and FLAG-EMP1 (Red) plasmids were co-transfected into Cos7 cells. Localization of expressed molecules was examined by confocal microscopy after counterstaining with DAPI. Green and red arrowheads indicate EGFP-copine-III and FLAG-EMP1 at the plasma membrane, respectively. Scale bar, 10 μm. **f** Association of FLAG-EMP1 with EGFP-copine-III in the membrane fraction. The membrane fraction of Cos7 cells transfected with FLAG-EMP1 and EGFP-copine-III was isolated by the kit and immunoprecipitated with an anti-FLAG antibody or control IgG, followed by western blotting with indicated antibodies. LDL receptor: a marker for the membrane fraction. **g** Association of FLAG-EMP1 with endogenous copine-III. Cell lysates of LNCaP #2 and #17 cells were immunoprecipitated with an anti-FLAG antibody, followed by western blotting with indicated antibodies. **h** Determination of the copine-III domain necessary for association with the intracellular region of EMP1. The expression vector of each mutant of EGFP-copine-III was transfected into HEK293 cells, and the cell lysates were pulled down with the control or peptide-bound beads used in **a**, followed by western blotting

We sought to identify which molecule(s) binds to the intracellular region of EMP1 to examine the downstream signaling of EMP1 responsible for the enhancement of cell migration. For this purpose, we used LNCaP cell lysates and Sepharose-beads bound with or without chemically synthesized peptides referred to the intracellular loop region of EMP1. Copine-III was identified by mass spectrometry as a protein specifically pulled down with the peptide-bound beads (Fig. 4a, arrow; Supplementary Figure S1). Copine-III is a 60-kDa protein with two N-terminal C2 domains (C2-1 and C2-2) followed by a von Willebrand A-like domain (VWA) (Fig. 4b) [17, 18]. We subsequently cloned the copine-III cDNA to produce the EGFP-tagged copine-III expression vector for transfection into the mammalian cells, and found that endogenous copine-III as well as EGFP-copine-III were pulled down with the EMP1-derived peptide-bound beads in HEK293 cells (Fig. 4c). FLAG-EMP1 was also pulled down with glutathione beads binding glutathione *S*-transferase (GST)-tagged copine-III (Fig. 4d). We next used immunostaining to observe the co-localization of EMP1 with copine-III at the cell surface following the transfection of both FLAG-EMP1 and EGFP-copine-III expression vectors into Cos7 cells (Fig. 4e). The co-localization was further confirmed by the immunoprecipitation experiment using the membrane fraction of Cos7 cell lysates (Fig. 4f). Endogenous copine-III was associated with FLAG-EMP1 in LNCaP #17 cells and endogenous EMP1 in PC3 cells (Fig. 4g; Supplementary Figure S2a). To obtain more information about the association of EMP1 with copine-III, copine-III mutants were transfected into HEK293 cells and the pull-down assay using EMP1-derived peptide-bound beads was conducted. Copine-III-ΔC2-1 was pulled down with the peptide-bound beads, but copine-III-ΔC2-2 and -ΔVWA were not (Fig. 4h), indicating that the C2-2 and VWA domains are necessary for the EMP1–copine-III interaction.

### Involvement of copine-III in EMP1 promoted cell migration

**Fig. 5:**
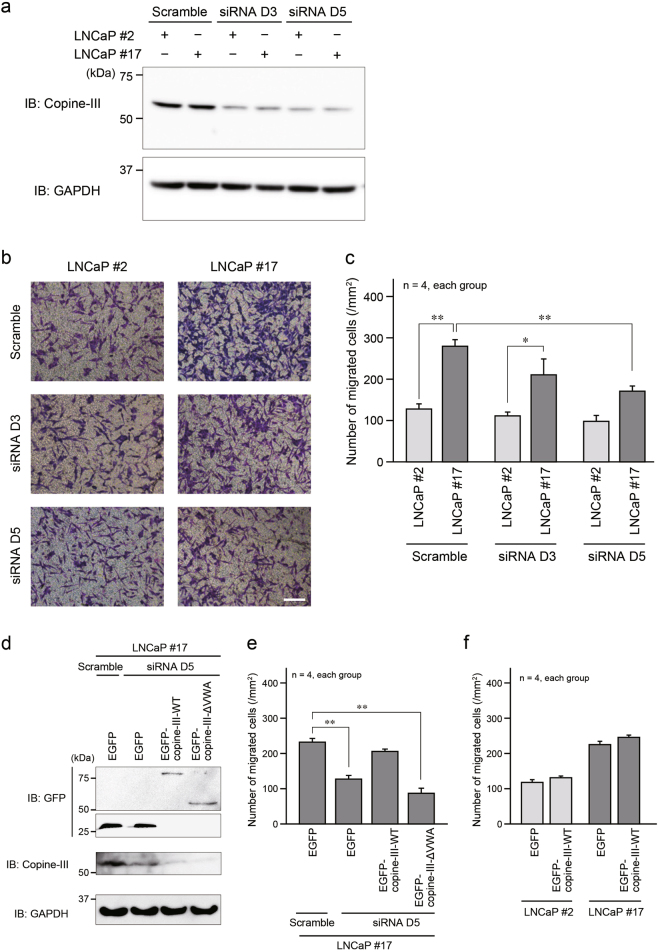
Effect of copine-III on EMP1 induced increase in cell migration. **a** Knockdown of copine-III. Two siRNAs (D3 and D5) for copine-III were applied to LNCaP #2 and #17 cells, and the cell lysates were used for western blotting. GAPDH: loading control. **b** Representative results of the Boyden chamber assay. Scale bar, 200 μm. **c** Summary graph of migrated LNCaP cells in the Boyden chamber assay. *p < 0.05 and **p < 0.01. **d** Expression levels of endogenous copine-III and transfected EGFP, EGFP-copine-III-WT or -ΔVWA in copine-III-knockdown LNCaP #17 cells. **e** Summary graph of migrated LNCaP #17 cells after knockdown of copine-III with or without re-expression of EGFP-copine-III-WT or -ΔVWA in the Boyden chamber assay. **p < 0.01. **f** Summary graph of migrated LNCaP #2 and #17 cells transfected with EGFP or EGFP-copine-III-WT in the Boyden chamber assay

Two siRNAs against copine-III (siRNA D3 and D5) were used to inhibit its expression in LNCaP cells, and siRNA D5 more effectively silenced copine-III than siRNA D3 (Fig. 5a). As shown in Fig. 3c, d, cell migration ability was higher in LNCaP #17 cells than LNCaP #2 cells in the Boyden chamber assay, and the ability of LNCaP #17 cells to migrate was significantly reduced following transfection of siRNA D5 (Fig. 5b, c). In a rescue experiment, EGFP, EGFP-copine-III-WT, which is resistant to siRNA D5, or EGFP-copine-III-ΔVWA, which is incapable of binding to EMP1, was transfected into copine-III-knockdown LNCaP #17 cells (Fig. 5d). The expression of EGFP-copine-III-WT almost fully recovered the reduction in cell migration observed when copine-III was suppressed, while the expression of EGFP-copine-III-ΔVWA did not (Fig. 5e). In LNCaP #2 cells, overexpression of EGFP-copine-III-WT did not increase cell migration (Fig. 5f). These results suggest that the association of EMP1 with copine-III is required to promote prostate cancer cell migration.

### Rac1 activation by EMP1 and copine-III

**Fig. 6:**
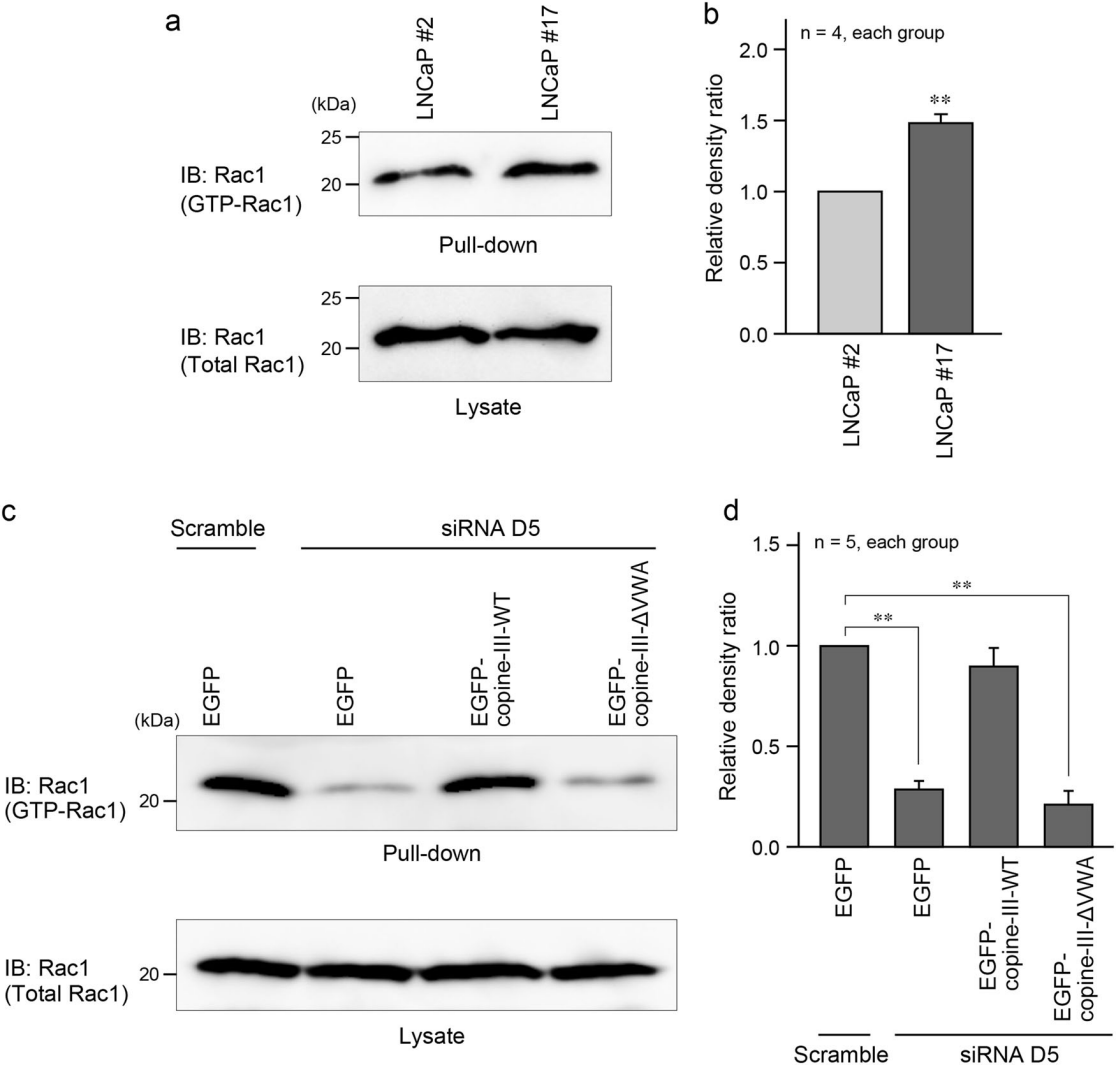
EMP1 induced activation of Rac1. **a, c** GTP-bound active form of Rac1 in each type of LNCaP cells was pulled down using beads bound to GST-PAK-CRIB, followed by western blotting. **b, d** Summary graph of the relative density ratio of GTP-bound Rac1 normalized to total Rac1. The value of the relative density ratio in LNCaP #2 **(b)** and scramble RNA- and EGFP-transfected LNCaP #17 **(d)** was set at 1.0. **p < 0.01 vs. LNCaP #2 in **b**. **p < 0.01 in **d**

To determine the underlying mechanism by which EMP1 and copine-III promote cell migration, we examined Rac1 activity by the pull-down assay using glutathione beads bound to GST-p21-activated kinase1 (PAK)-Cdc42 and Rac-binding domain (CRIB), as Rac1 essentially activates cell migration and cancer metastasis [19]. We found that in the assay, the amount of GTP-bound activated Rac1 was increased in LNCaP #17 cells compared with LNCaP #2 cells (Fig. 6a, b). Moreover, in LNCaP #17 cells, Rac1 activation was attenuated by copine-III knockdown and rescued by the expression of EGFP-copine-III-WT, but not EGFP-copine-III-ΔVWA (Fig. 6c, d).

### Molecular mechanism of EMP1-induced Rac1 activation

**Fig. 7:**
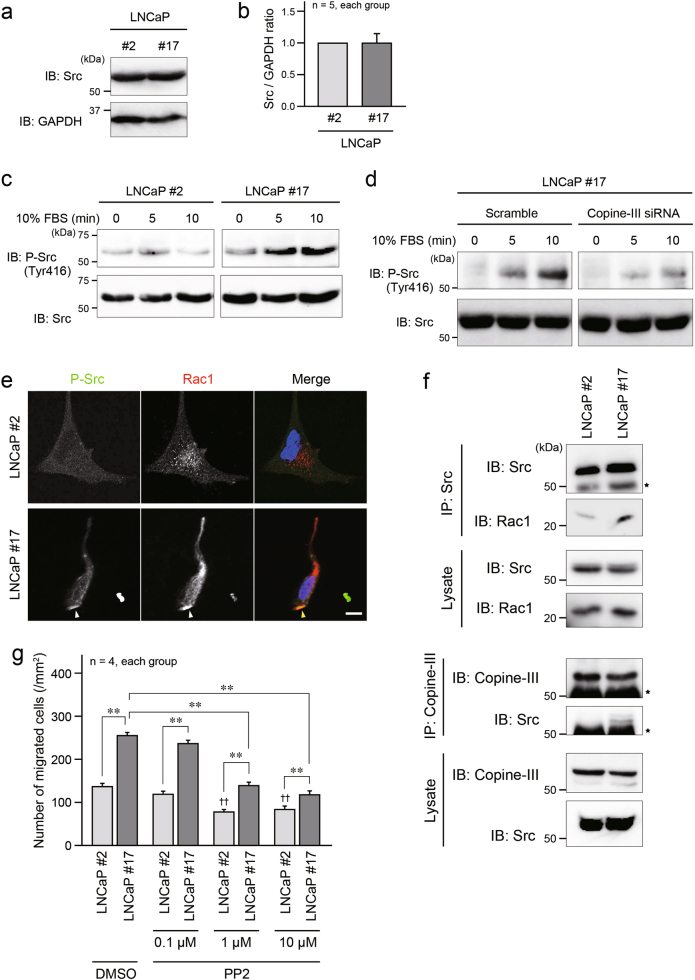
Significance of Src activation for EMP1 induced increase in cell migration. **a** Expression level of Src in LNCaP #2 and #17 cells. **b** Summary graph of the relative density ratio of Src normalized to GAPDH. The value of the relative density ratio in LNCaP #2 was set at 1.0. **c** EMP1 enhanced the phosphorylation of Src (P-Src). After overnight serum starvation, LNCaP cells were stimulated by fetal bovine serum (FBS) for the indicated durations. **d** Involvement of copine-III in EMP1-induced Src activation. LNCaP #17 cells transfected with copine-III siRNA D5 or scramble as a control were treated with or without FBS for the indicated durations after overnight serum starvation. **e** Co-localization of phosphorylated Src with Rac1 at the protrusive cell periphery of LNCaP #17 cells (arrowheads) was observed in confocal microscopy. Blue: DAPI. Scale bar, 10 μm. **f** Increased association of Src with Rac1 and copine-III in the presence of EMP1. Cell lysates of LNCaP #2 and #17 cells were immunoprecipitated with an anti-Src (upper panels) or an anti-copine-III (lower panels) antibody, followed by western blotting with indicated antibodies. Star: IgG heavy chain. **g** Summary graph of migrated LNCaP #2 and #17 cells in the Boyden chamber assay in the presence of DMSO (control) or PP2 (Src inhibitor). **p < 0.01, and ^††^*p* < 0.01 vs. LNCaP #2 cells treated with DMSO

The activation of Rho family small G proteins, including Rac1, is mainly regulated by guanine nucleotide exchange factors (GEFs), which increase the GTP-bound active form, and GTPase-activating proteins, which inactivate Rac1 by hydrolyzing GTP to GDP [20, 21]. One of the Rac1-GEFs is Vav2 and its activity is upregulated by Src-mediated phosphorylation [22, 23]. Because Src is reported to be activated downstream of copine-III [24], we examined whether the EMP1–copine-III axis could contribute to the increase in Src activity in prostate cancer cells. The expression level of Src was similar between LNCaP #2 and #17 cells (Fig. 7a, b). In LNCaP #17 cells, activated Src, which was detected by the phosphorylation of Tyr416, was increased by stimulation with serum in a time-dependent manner, while in LNCaP #2 cells, little Src activation was observed (Fig. 7c). The Src activation in LNCaP #17 cells was dependent on copine-III (Fig. 7d). Activated Src accumulated at the cell protrusion of motile LNCaP #17 cells, and was co-localized with Rac1 (Fig. 7e). This was confirmed by the immunoprecipitation experiment, which also showed the association between copine-III and Src (Fig. 7f). In addition, the association of Src with copine-III and Rac1 was detected in PC3 cells (Supplementary Figure S2b). When the activation of Src was inhibited by pretreatment with a Src inhibitor PP2, LNCaP cell migration was significantly suppressed in a PP2 concentration-dependent manner, compared with pretreatment using DMSO as a control (Fig. 7g). The PP2-mediated reduction of cell migration was more remarkable in LNCaP #17 than LNCaP #2 cells, suggesting the importance of Src activation for EMP1-induced cell migration.

**Fig. 8:**
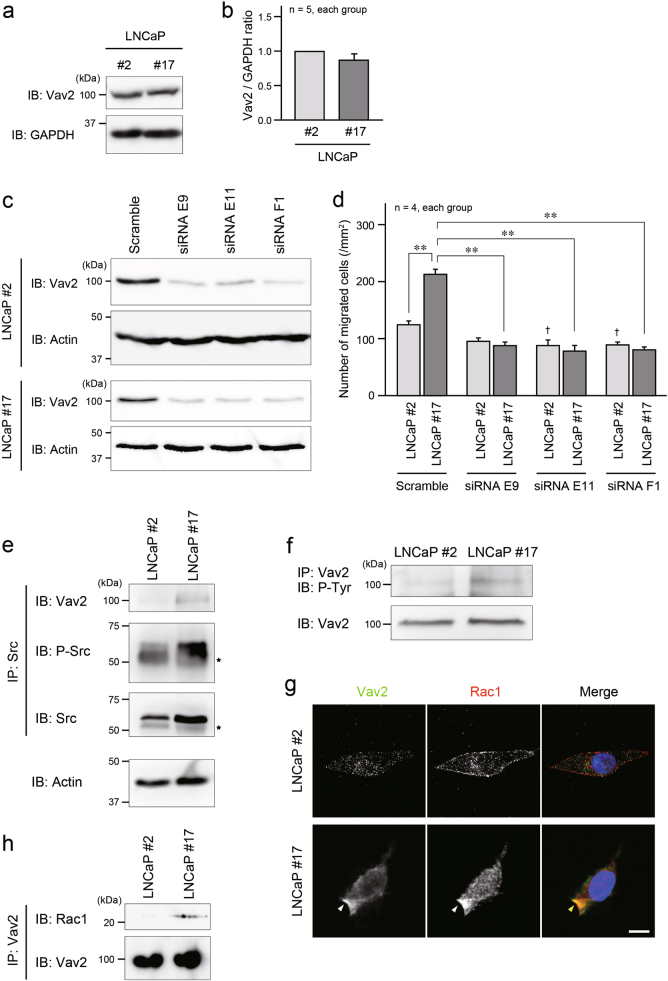
EMP1-induced activation of Vav2, a Rac1-GEF, for cell migration. **a** Expression level of Vav2 in LNCaP #2 and #17 cells. **b** Summary graph of the relative density ratio of Vav2 normalized to GAPDH. The value of the relative density ratio in LNCaP #2 was set at 1.0. **c** Knockdown of Vav2. Three siRNAs (E9, E11, and F1) for Vav2 were applied to LNCaP cells, and the cell lysates were used for western blotting. Actin: loading control. **d** Summary graph of migrated LNCaP cells in the Boyden chamber assay. **p < 0.01, and ^†^*p* < 0.05 vs. LNCaP #2 cells with scramble RNA. **e** Association of Vav2 with Src in the presence of EMP1. Cell lysates of LNCaP cells were immunoprecipitated with an anti-Src antibody, followed by western blotting with indicated antibodies. Actin: loading control. Star: IgG heavy chain. **f** Activation of Vav2 by EMP1. Cell lysates of LNCaP cells were immunoprecipitated with an anti-Vav2 antibody, followed by western blotting with an antibody to specifically detect the phosphorylated tyrosine residue (P-Tyr). **g** Co-localization of Vav2 with Rac1 at the cell periphery of LNCaP #17 cells (arrowheads) was observed in confocal microscopy. Blue: DAPI. Scale bar, 10 μm. **h** Increased association of Vav2 with Rac1 in the presence of EMP1. Cell lysates of LNCaP cells were immunoprecipitated with an anti-Vav2 antibody, followed by western blotting with Vav2 and Rac1 antibodies

To determine the relationship between Vav2 and EMP1 in prostate cancer cells, we first confirmed that Vav2 expression levels were similar between LNCaP #2 and #17 cells (Fig. 8a, b). Then, we knocked down Vav2 in LNCaP #2 and #17 cells. Three siRNAs against Vav2 (siRNA E9, E11, and F1) reduced the expression of Vav2 to a similar extent (Fig. 8c). In LNCaP #17 cells, the Vav2 siRNAs significantly attenuated cell migration, compared with the control siRNA (Scramble), and the inhibitory effect of Vav2 siRNAs on cell migration was more remarkable in LNCaP #17 cells than LNCaP #2 cells (Fig. 8c, d). The association of Vav2 with Src was observed in LNCaP #17 cells and PC3 cells by immunoprecipitation (Fig. 8e; Supplementary Figure 2b). The activation of Vav2, which was determined by its phosphorylation, was higher in LNCaP #17 cells than in LNCaP #2 cells (Fig. 8f). The co-localization of Vav2 with Rac1 was also detected at the cell periphery of motile LNCaP #17 cells (Fig. 8g), and the association between Vav2 and Rac1 in LNCaP #17 cells was observed by immunoprecipitation (Fig. 8h). Taken together, we identified that Src and Vav2 activation mediated the EMP1-induced enhancement of Rac1 activity and cell migration, concomitant with copine-III.

### Role of EMP1 in other cancer cell lines

**Fig. 9:**
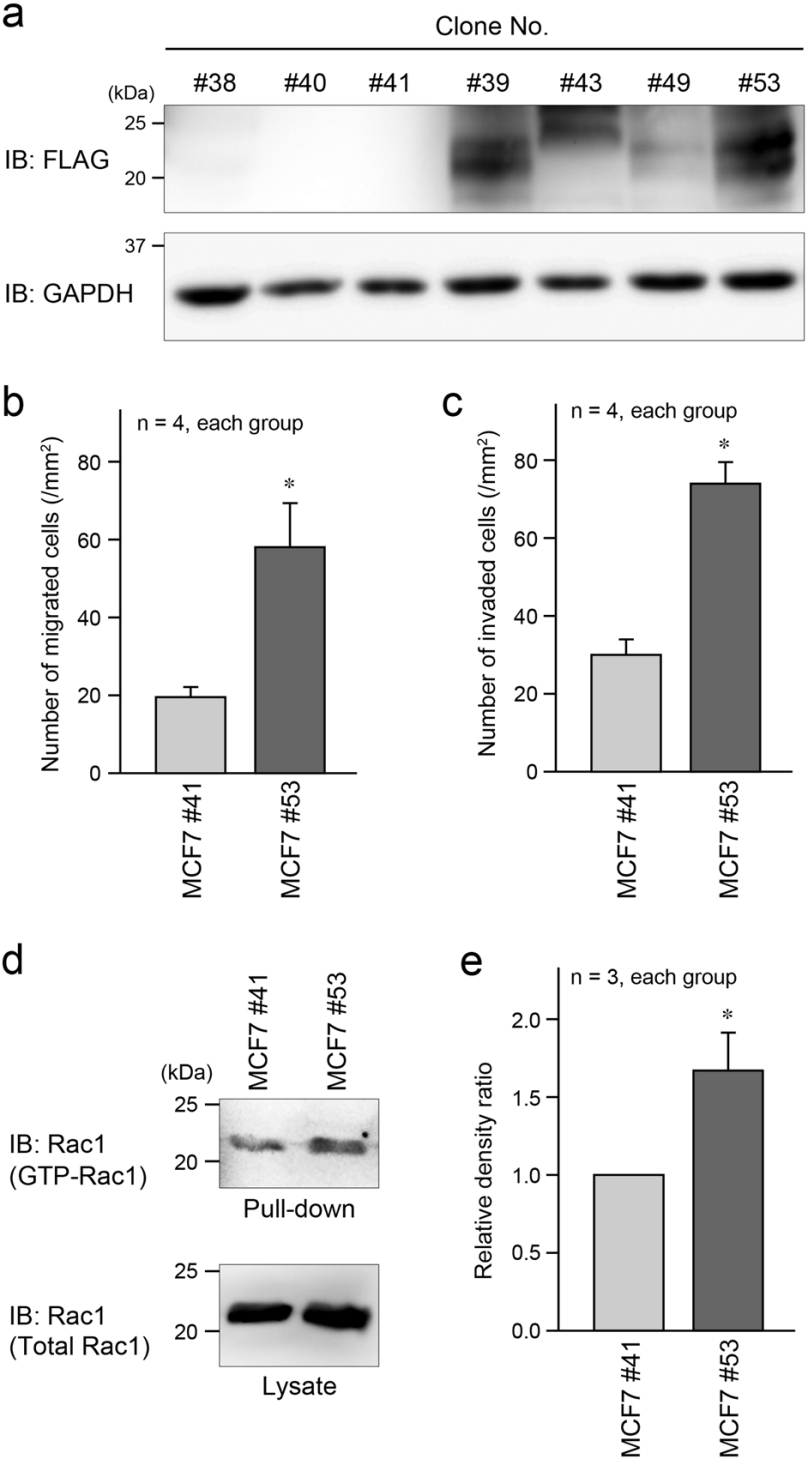
EMP1 induced increase in cell migration, invasion, and Rac1 activation in MCF7 breast cancer cells. **a** Several MCF7 clones stably expressing FLAG-EMP1 were verified by western blotting. **b** Summary graph of migrated MCF7 cells in the Boyden chamber assay. MCF7 cells with subtle expression (clone #41) and high expression (clone #53) of FLAG-EMP1 were assessed in this experiment. *p < 0.05 vs. MCF7 #41. **c** Summary graph of migrated MCF7 cells in the invasion assay. *p < 0.05 vs. MCF7 #41. **d** GTP-bound active form of Rac1 in each type of MCF7 cells was pulled down using beads bound to GST-PAK-CRIB, followed by western blotting. **e** Summary graph of the relative density ratio of GTP-bound Rac1 normalized to total Rac1. The value of MCF7 #41 was set at 1.0. *p < 0.05 vs. MCF7 #41

In addition to LNCaP cells, we used human breast cancer MCF7 cells to more broadly certify the pathological role of EMP1 in cancer. As with LNCaP cells, we generated clones of MCF7 cells stably expressing FLAG-EMP1 (Fig. 9a), and selected MCF7 #41 cells with subtle expression of FLAG-EMP1, and MCF7 #53 cells with abundant FLAG-EMP1 expression for the following experiments. Consistent with the results obtained in LNCaP cells, cell migration in the Boyden chamber and invasion assays and Rac1 activation in the pull-down assay were significantly higher in MCF7 #53 cells than in MCF7 #41 cells (Fig. 9b−e). Similar results were also obtained in colon cancer Caco-2 cells that highly and faintly expressed FLAG-EMP1 (Caco-2 #26 and Caco-2 #24, respectively) (Supplementary Figure S3).

### Significance of EMP1 expression for human cancer progression

**Fig. 10:**
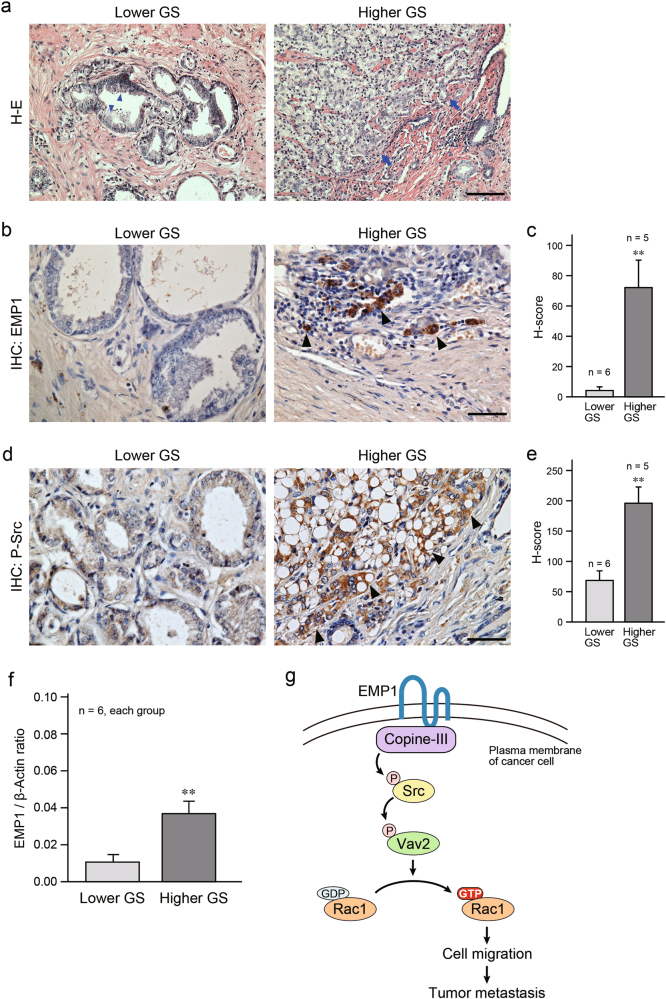
Correlation between EMP1 expression and malignancy of human prostate cancer. **a** Representative H–E staining of human prostate cancer samples with lower Gleason score (GS; 6 or 7) or higher Gleason score (8 or 9). Arrowheads: accumulation of cancer cells; Arrows: invasive cell front. **b, d** Representative results of immunohistochemistry for EMP1 and P-Src expression. Nuclei were counterstained with hematoxylin. Arrowheads: EMP1- or P-Src-positive cancer cells. **c, e** Summary graph of *H*-score. **p < 0.01 vs. lower Gleason score. **f** Summary graph of qPCR results in samples with lower or higher Gleason score. Relative mRNA expression of *EMP1* was normalized to that of *β-actin*. **p < 0.01 vs. lower Gleason score. **g** Schematic illustration of EMP1-induced signal transduction in cancer cells. Scale bars, 100 μm in **a** and 50 μm in **b,** **d**

Finally, we analyzed the expression of EMP1 in human prostate cancer samples. Their pathological characteristics are indicated in Supplementary Table S3. Six and five samples from lower (LGS-1 through LGS-4) and higher (HGS-1 through HGS-4) Gleason score groups, respectively, were used in the staining experiments. Hematoxylin and eosin (H–E) staining showed that crowded, small, prostate glands of irregular in size and shape were observed in samples from patients with lower Gleason score (Fig. 10a, left). Moreover, in the higher Gleason score samples, prostate glands were virtually absent due to filling of cancer cells in the glands, and the tumors had invaded the surrounding connective tissues (Fig. 10a, right). Immunohistochemistry showed strong EMP1-positive staining in the prostate tumor areas, especially at the invasive front, of the higher Gleason score samples compared with the lower Gleason score samples (Fig. 10b, c). Similar staining patterns were observed for phosphorylated Src (Fig. 10d, e). In quantitative PCR (qPCR) analysis, expression of EMP1 was elevated in the higher Gleason score samples (Fig. 10f). These results suggest that increased expression of EMP1 may be related to malignancy that leads to metastasis, and may therefore represent a novel prognostic marker for prostate cancer.

## Discussion

This is the first study to provide evidence that the transmembrane protein EMP1 potentiates tumor metastasis in a mouse model via an underlying mechanism of Rac1 activation that in turn is responsible for promoting the invasive migration of cancer cells. EMP1-related potentiation of tumor metastasis is supported by the finding that prostate cancer samples with higher Gleason score abundantly express EMP1, compared with those with lower Gleason score. Considering not a large sample size of the experiment, the continuous attempt to increase the human prostate cancer samples for analyzing them would be necessary to strengthen the clinical significance of EMP1 expression in metastasis and poor prognosis of the prostate cancer. As the upregulation of EMP1 in prostate cancer cells was identified by the interaction between cancer cells and stromal cells relevant to the tumor microenvironment, which is not fully elucidated but considered to be important for tumor metastasis [25, 26], our study may provide a new insight into understanding of tumor progression and metastasis.

EMP1 belongs to the peripheral myelin protein 22-kDa gene family that encompasses proteins with tetra-spanning membrane domains [27], and has a high degree of domain structure homology with EMP2 and EMP3 [28]. Although EMP1 is reportedly downregulated in the cancer region as compared with the adjacent normal epithelium at the primary tumor site [29], this molecule has also been proposed as a marker of resistance to cancer therapies and related to poor prognosis [30, 31]. The latter studies support our conclusion that EMP1 functions as a progressive and pro-metastatic factor in cancer. Prospective clinical studies in the future may provide a certain evidence that EMP1 expression is related to prognosis of prostate cancer. Besides EMP1, EMP2 has been shown to be upregulated in more than 70% of both serous and endometrioid ovarian cancers [32], and to enhance tumor growth of glioblastoma by promoting angiogenesis, partially by increasing vascular endothelial growth factor-A expression in glioblastoma cells [33, 34]. EMP3 functionality appears to be related to cancer type. EMP3 promotes cancer cell proliferation and growth in urothelial carcinomas [35], while it is considered to be a tumor suppressor in other cancers [36, 37]. Other tetra-spanning membrane proteins also appear to be involved in the determination of cancer phenotype. Claudin-20, for example, a tight junction protein in the epithelium, is correlated with poor prognosis of breast cancer patients, likely through its increased migratory effect on cancer cells [38]. CD151 is correlated with poor prognosis in endometrial cancer [39], accelerates breast cancer cell invasion via integrin α6 [40], and promotes prostate cancer migratory activity and lymphangiogenesis, increasing metastatic potential [41]. Based on these studies and our findings, targeting of the tetra-spanning membrane proteins, including EMP1, may reduce the malignancy of cancer cells, leading to the development of novel anti-cancer therapeutics.

We further demonstrated that copine-III plays a role downstream of EMP1 to promote prostate cancer metastasis. The copine family consists of seven members that bind to phospholipid in the plasma membrane in a Ca^2+^-dependent manner [18]. Although its enzymatic activity has not been examined, copine-III is reported to function in membrane trafficking [42]. Genome-wide association analysis shows that an SNP in the *copine-III* gene is associated with susceptibility to prostate cancer [43]. Analysis using microarray data sets from the ONCOMINE database indicates that the expression of copine-III is significantly upregulated in metastatic prostate cancer and ovarian endometrioid adenocarcinoma, compared with normal tissues [24, 44]. In breast cancer cells, copine-III binds to the tyrosine kinase receptor ErbB2 (HER2) in a phosphorylation-dependent manner to activate Src for cancer cell migration [24]. In agreement with these previous studies, copine-III was shown to be essential for EMP1-induced prostate cancer cell migration via the activation of the Src–Vav2–Rac1 axis by the loss-of-function and subsequent rescue experiments in our study.

With respect to clinical implications, development of an inhibitory antibody to specifically target EMP1 to infer the potential of this molecule may be challenging because of the high expression of EMP1 in pro-metastatic cancers. To date, immunotherapy based on monoclonal antibodies against cancer cell surface proteins, including those with four transmembrane domains (like EMP1), has been clinically approved and shown to be effective for the treatment of several types of cancer [45]. These antibodies can directly or indirectly exhibit cytotoxicity to cancer cells by an antibody-dependent perturbation of the target molecule or by complement-mediated effects. The intracellular loop region of EMP1 also represents a candidate target to inhibit cancer metastasis, because EMP1-induced signal transduction is triggered by the complex formation of EMP1 and copine-III. For example, the nine amino acid peptide (FTMEKGNRF) at the intracellular loop of EMP1, which was used in the identification of copine-III and binds to copine-III, may block the EMP1–copine-III binding and thus limit the effects of EMP1 on cancer metastasis. To allow such peptides to pass into the cell, the addition of TAT (a cell penetrating sequence of human immunodeficiency virus) to the peptide can be utilized [46].

Taken together, this study describes a potential mechanism of cancer metastasis via EMP1 signaling. EMP1 and its binding partner copine-III facilitate the activation of Src, Vav2, and Rac1, resulting in enhanced cancer cell migration and metastasis as summarized in Fig. 10g. Targeting of EMP1 may have anti-cancer therapeutic potential by inhibiting cancer metastasis to reduce mortality.

## Materials and methods

### Cell culture and animals

LNCaP cells, MCF7 cells, MDA-MB-231 cells, Caco-2 cells, HEK293 cells, and Cos7 cells were cultured in Dulbecco’s Modified Eagle Medium (DMEM) (Nacalai Tesque, Kyoto, Japan); PC3 cells were cultured in RPMI 1640 medium (Nacalai Tesque); and A375 cells were cultured in Eagle’s Minimum Essential Medium (Wako Pure Chemical Industries, Osaka, Japan). All media were supplemented with 10% fetal bovine serum (FBS) (Sigma-Aldrich, Saint Louis, MO, USA), 20 mM l-glutamine (Nacalai Tesque) and 100 µ/mL penicillin–streptomycin (Nacalai Tesque). PrS cells were purchased from Lonza (Cat. No. CC-2508; Basel, Switzerland) and cultured in Stromal cell basal medium supplemented with growth factors (Lonza). Male BALB/c nude mice (7-weeks-old) were purchased from Japan SLC (Shizuoka, Japan). The animal experiments conducted in this study were approved by the Shiga University of Medical Science Animal Care and Use Committee according to the Animal Research Reporting of In Vivo Experiments guidelines..

### Mammalian expression vectors and cells stably expressing EMP1

Full-length cDNAs of human EMP1 and copine-III were obtained by reverse transcription PCR using RNA extracted from human cultured cells. EMP1 cDNA was subcloned into the pFLAG-CMV5 vector (Sigma-Aldrich), and then EMP1 cDNA including the FLAG-tag sequence was cut out and inserted in the pcDNA3.1-Hygro vector (Thermo Fisher Scientific, Waltham, MA, USA) to create the pcDNA3.1-Hygro-EMP1-FLAG vector. EGFP-tagged copine-III-WT (amino acids 1–537), copine-III-ΔC2-1 (amino acids 139–515), copine-III-ΔC2-2 (amino acids 7–125 and 263–515), and copine-III-ΔVWA (amino acids 7–251) were constructed with the pEGFP-C1 vector (BD Biosciences, San Jose, CA, USA). A siRNA-resistant vector for EGFP-copine-III-WT was created by alteration of three nucleotides in the siRNA-target sequence by the QuikChange site-directed mutagenesis kit (Agilent, Santa Clara, CA, USA). All sequences inserted into vectors were confirmed by ABI Prism 3100xl Genetic Analyzer (Applied Biosystems, Waltham, MA, USA). To generate LNCaP, MCF7 and Caco-2 cells stably expressing EGFP or FLAG-EMP1, the pEGFP-C1 or pcDNA3.1-Hygro-EMP1-FLAG vector was transfected into these cells, and the cells were then cultured in media containing 400 μg/mL G418 (Nacalai Tesque) or hygromycin B (Nacalai Tesque), respectively. For cell proliferation assays, cells were plated at 1 × 10^4^ cells/well in 24-well plates. Trypsinized cells were counted using a cell counting chamber (Erma, Tokyo, Japan) at the indicated time points.

### Co-culture of LNCaP cells with PrS cells for gene-expression analysis

EGFP-labeled LNCaP cells were co-cultured with the same number of PrS cells for 48 h, or cultured alone as a control. Cell culture-conditioned media were mixed between dishes every 6 h. After the co-culture, EGFP-labeled LNCaP cells were isolated from PrS cells by flow cytometry (FACSAria; BD Biosciences). RNA was extracted from LNCaP cells using High Pure RNA Isolation Kit (Roche Diagnostics, Mannheim, Germany). Gene expression levels were analyzed with Affymetrix DNA microarray chips (Thermo Fisher Scientific), and were compared between control and co-cultured LNCaP cells.

### Identification of proteins that interact with the intracellular region of EMP1

A chemically generated peptide consisting of nine amino acids (FTMEKGNRF) referred to the intracellular loop region of human EMP1 was immobilized onto CNBr-activated Sepharose 4B beads (GE Healthcare, Piscataway, NJ, USA). The beads coated with the peptide or uncoated were resuspended in LNCaP cell lysates for 16 h at 4 °C. Subsequently, proteins bound to the beads were subjected to sodium dodecyl sulfate-polyacrylamide gel electrophoresis (SDS-PAGE), followed by Coomassie Brilliant Blue staining for protein visualization. Following the excision of protein bands from the gel, they were destained and the proteins were digested by sequence-grade modified trypsin (Promega, Madison, WI, USA). The cleaved peptides were purified and analyzed by tandem mass spectrometry (Finigan LCQ Advantage MAX and Xcalibur Bioworks v.3.2; Thermo Electron, Waltham, MA, USA) to identify the protein sequences. Full-length human copine-III cDNA was subcloned into the pGEX-6P1 vector (GE Healthcare), and GST-copine-III was expressed in *E. coli* transformed with the vector. After sonicating the cells, GST-copine-III was purified from the cell lysates using glutathione-Sepharose 4B beads (GE Healthcare) as described previously [47].

### Implantation of LNCaP cells in nude mice

Control (parental) or EMP1-overexpressing LNCaP cells were resuspended in 50 µL of a 1:2 mixture of serum-free DMEM and Matrigel (BD Biosciences) at 1 × 10^7^ cells, and implanted subcutaneously or orthotopically into the prostate of male nude mice aged 7–8 weeks. Tumor growth after subcutaneous injection was monitored for 12 weeks, and tumor size was measured by a digital caliper. Tumor volume was calculated as follows: Volume (mm^3^) = 0.52 × (short axis [mm])^2^ × long axis (mm). Six weeks after orthotopic injection into the prostate, mice were sacrificed for histopathological analysis.

### siRNA transfection

Copine-III siRNA, Vav2 siRNA, and negative control RNA (Scramble) were purchased from Thermo Fisher Scientific. siRNA sequences against copine-III (siRNA D3 and D5) and Vav2 (siRNA E9, E11 and F1) were as follows: copine-III siRNA D3 5′-UUCAAAUUCAACAGGUGAGCUUCUG-3′, copine-III siRNA D5 5′-CAACAGCAGACAGCUUCUCAAUAUU-3′, Vav2 siRNA E9 5′-AGGUAUAACUUUGCCGCCCGAGAUA-3′, Vav2 siRNA E11 5′-GCGGACAUGGCAGCUGUCUUCAUUA-3′, and Vav2 siRNA F1 5′-CAGCGAAUUUCAGAGUUCUAUAGAA-3′.

### Boyden chamber and invasion assays

Cells were seeded at 5 × 10^4^ cells/well in the upper chamber of Transwell chambers coated with 1 µg of fibronectin for the Boyden chamber assay and with 100 μL of 0.15 mg/mL Matrigel for the invasion assay. Pretreatment with PP2 (1 μM; Merck Millipore, Billerica, MA, USA) or DMSO (Nacalai Tesque) was conducted for 30 min before starting the assay. DMEM supplemented with 10% FBS was placed in the lower chamber as a chemoattractant, and cells were allowed to migrate for 16 h. Cells that had migrated on the lower surface of the chamber were stained using Diff-Quik Kit (Sysmex, Kobe, Japan) to count the number of viable cells.

### Protein extraction, immunoprecipitation, and western blotting

Total cell lysates were obtained using RIPA lysis buffer (50 mM Tris-HCl [pH 7.5], 150 mM NaCl, 0.5% deoxycholate sodium, 0.1% SDS, 1% Nonidet P-40, and 1 mM phenylmethylsulfonyl fluoride). Protein deglycosylation mix kit (Promega) was used to detect glycosylation of EMP1. Cell surface proteins were isolated and collected by Mem-PER^TM^ Plus Membrane Protein Extraction Kit (Thermo Fisher Scientific). For immunoprecipitation, supernatants were incubated with primary antibody (1:100 dilution) overnight at 4 °C. Next, 15–20 µL of protein G Sepharose beads were added and samples were incubated for 1 h at 4 °C. Proteins bound to the beads were clarified by centrifugation at 8000 × *g* for 2 min at 4 °C, washed with the lysis buffer, eluted in the loading buffer, and used in western blotting. The list of primary antibodies is provided in Supplementary Table S4. A rabbit polyclonal anti-EMP1 antibody was originally generated in our laboratory using a synthetic peptide (YTSHYANRDGTQYHHGYS: amino acids 116–133 of EMP1) as an antigen. Incubation with peroxidase-conjugated secondary antibody (anti-mouse/rabbit 1:5000 dilution; GE Healthcare) was performed for 1 h at room temperature. Finally, protein expression was visualized using a chemiluminescence substrate reagent and quantified by ImageJ software (National Institutes of Health, Bethesda, MD, USA).

### Pull-down assay for Rac1

This experiment was conducted as previously described [22]. Briefly, protein lysates were incubated with 15 µg of GST-PAK-CRIB protein-bound glutathione beads for 1 h at 4 °C. Next, the lysates were centrifuged for 1 min at 5000 × *g*, 4 °C, and the supernatants were removed without disturbing the beads. Beads were then washed with the lysis buffer and loading buffer was added. Samples were analyzed by western blotting to detect GTP-bound activated Rac1.

### Immunofluorescence staining

Immunofluorescence staining experiments were conducted as previously described [48, 49]. For cell staining, cells grown on glass coverslips coated with fibronectin were fixed with 4% paraformaldehyde (PFA) and permeabilized with 0.2% Triton X-100 for 5 min. For tissue staining, cryosections were fixed in 4% PFA, washed with PBS for 15 min, and permeabilized with 0.3% Triton X-100 for 5 min. Nonspecific staining was reduced by blocking with 3–5% bovine serum albumin for 30 min at room temperature. Samples were incubated overnight at 4 °C with primary antibody at appropriate dilutions. The secondary fluorescent antibodies Alexa Fluor 488 and Alexa Fluor 555 (1:200–1000 dilution) (Thermo Fisher Scientific) were applied for 1 h at room temperature in the dark. After washing with PBS, nuclei were stained with DAPI (1:200 dilution) (Dojindo; Dojindo, Kumamoto, Japan) for 5 min.

### Chromogenic staining

Six lower Gleason score (score 6 or 7; LGS-1 through LGS-4 in Supplementary Table S3) samples and five higher Gleason score (score 8 or 9; HGS-1 through HGS-4) samples were used in this experiment. Endogenous peroxidase activity in all sections was quenched for 30 min using 3% hydrogen peroxidase (H_2_O_2_) prior to overnight incubation with a rabbit polyclonal anti-EMP1 or anti-P-Src antibody (1:200 dilution). After washing with PBS, samples were incubated with a biotin-conjugated anti-rabbit IgG (H+L) secondary antibody (1:200 dilution) (Thermo Fisher Scientific) for 1 h at room temperature, followed by incubation with the streptavidin-peroxidase kit (Nacalai Tesque) for 30 min to form the avidin–biotin complex, which was detected with the Strep ABC peroxidase kit (Nacalai Tesque). Samples were counterstained with hematoxylin, followed by dehydration steps. The quantification of chromogenic staining was evaluated by the *H*-score, which is based on the predominant staining intensity of DAB in tumor cells (0, negative; 1+, weak; 2+, moderate; 3+, intense) [50]. The total number of cells and the positively stained tumor cells at each intensity located at the invasive front were calculated and percentage of the cells were obtained. The *H*-score ranging from 0 to 300 was calculated using the following formula: *H*-score = 1 × (percentage of score 1 cells)+2 × (percentage of score 2 cells)+3 × (percentage of score 3 cells).

### Total RNA extraction from formalin-fixed paraffin-embedded human tissues

Formalin-fixed paraffin-embedded prostate cancer samples (*n* = 6 from each lower and higher Gleason score group) were sectioned and deparaffinized. Detailed pathological characteristics of samples are indicated in Supplementary Table S3. Total RNA was extracted from the samples using the ReliaPrep FFPE Total RNA Miniprep System (Promega). The experiments and procedures using human prostate cancer samples were approved by the Research Ethics Committee at Shiga University of Medical Science.

### Semi-quantitative PCR and qPCR

For semi-quantitative PCR, total RNA was extracted from cells of each cancer cell line using TRIzol reagent (Thermo Fisher Scientific), and cDNA was synthesized by ReverTra Ace qPCR RT Master Mix with gDNA Remover (Toyobo, Osaka, Japan). After 30 cycles of thermal cycling, band densities of amplified DNA fragments were measured using the ImageJ software. The primers used were as follows: *EMP1* (forward 5′-GCCAACATGTTGGTATTGCT-3′ and reverse 5′-TTATTTCTTTCTCAGGACCA-3′) and *GAPDH* (forward 5′-AGCCACATCGCTCAGACAC-3′ and reverse 5’-GCCCAATACGACCAAATCC-3’). qPCR was performed as previously described [47]. Total RNA extraction and cDNA synthesis were conducted as described above. The cDNA templates were further used in qPCR assay using the Thunderbird SYBR qPCR mix (Toyobo). *EMP1* (forward 5′-CCCTCCTGGTCTTCGTGT-3′ and reverse 5′-GGAATAGCCGTGGTGATA-3′) and *β-actin* (forward 5′-TCCTCCCTGGAGAAGAGCTA-3′ and reverse 5′-GAGTCCTGTGGCATCCAC-3′) gene primers were used in this experiment, and samples were run in duplicate in 96-well plates using LightCyler Instrument (Roche Diagnostics). The data were quantified by the standard curve method.

### Statistical analysis

Data are expressed as mean ± S.E.M. Experiments were performed at least three times independently. Statistical significance was analyzed using Prism 5 software (GraphPad Software, La Jolla, CA, USA) and determined by the unpaired two-tailed *t*-test, Fisher’s exact test, one-way ANOVA or two-way repeated measure ANOVA, as appropriate. If ANOVA was significant, individual difference was evaluated using the Bonferroni post-test. Sample sizes and statistical significance are indicated in the figures. A value of *p* < 0.05 was considered to be statistically significant. Sample sizes of sufficient power were chosen on the basis of similar published research and were confirmed statistically by appropriate tests. Experiments were not randomized and experimenters were not blinded to experimental conditions.

## Electronic supplementary material

Supplementary Information

Movie S1

Movie S2
