# Supplementary material for: Epithelial membrane protein 1 promotes tumor metastasis by enhancing cell migration via copine-III and Rac1
Source: Oncogene. 2018 Jun 4;37(40):5416–34. doi: 10.1038/s41388-018-0286-0 (PMC6172191; doi:10.1038/s41388-018-0286-0)
Supplement: Supplementary file 1 — Supplementary Information [file 41388_2018_286_MOESM1_ESM.pdf]

Supplementary information

Figure S1. Mass spectrometry of proteins bound to the peptide referred to the intracellular loop region of EMP1. Trypsin-digested proteins were fractioned with reverse phase liquid chromatography and analyzed with tandem mass spectrometry. The two marked signals were identical to the parts of human copine-III as indicated below.

Figure S2. Association of proteins related to the EMP1-mediated signaling in prostate cancer PC3 cells. **(a and b)** Cell lysates of PC3 cells were immunoprecipitated with an anti-copine-III **(a)** or anti-Src **(b)** antibody, followed by western blotting with indicated antibodies. Arrowhead: EMP1. Star: IgG heavy chain, and double star: IgG light chain.

Figure S3. EMP1-induced increase in cell migration and invasion in colon cancer Caco-2 cells. **(a)** Several Caco-2 clones stably expressing FLAG-EMP1 were verified by western blotting. **(b and c)** Summary graph of migrated Caco-2 cells in the Boyden chamber and invasion assays. Caco-2 cells with low expression (clone #24) and high expression (clone #26) of FLAG-EMP1 were assessed in these experiments. **, $p<0.01$ vs. Caco-2 #24.

Movie S1. Collective cell migration assay using LNCaP #2 cells. Confluent cells were cultured in serum-free DMEM overnight. Just after scratching the cell layer by a needle, culture media were changed to DMEM supplemented with 10% FBS and the time-lapse observation of cell migration was conducted by light microscopy for 12 h.

Movie S2. Collective cell migration assay using LNCaP #17 cells. The procedure was same as described in Movie S1.

Table S1. List of genes that were upregulated more than 3-fold in co-cultured LNCaP cells.

Gene name	Fold increase
Decorin (DCN)	26.85
Fibulin 1 (FBLN1)	23.08
Microfibrillar-associated protein 4 (MFAP4)	20.27
Collagen, type I, alpha 2 (COL1A2)	18.69
Collagen, type III, alpha 1 (COL3A1)	16.62
Transforming growth factor beta-induced (TGFBI)	15.08
Dickkopf homolog 3 (Xenopus laevis) (DKK3)	14.12
Secreted protein, acidic, cysteine-rich (Osteonectin) (SPARC)	13.62
TIMP metallopeptidase inhibitor 1 (TIMP1)	11.45
S100 calcium binding protein A6 (S100A6)	11.41
Lectin, galactoside-binding, soluble, 3 (LGALS3)	9.36
Glutathione S-transferase pi 1 (GSTP1)	7.94
Thymosin beta 4, X-linked (TMSB4X)	7.62
Nuclear receptor subfamily 2, group F, member 1 (NR2F1)	7.12
Follistatin-like 1 (FSTL1)	6.76
Stomatin (STOM)	5.09
Fibrillin 1 (FBN1)	4.99
Four and a half LIM domains 2 (FHL2)	4.95
Collagen, type VI, alpha 3 (COL6A3)	4.78
Annexin A2 (ANXA2)	4.19
Complement component 1, s subcomponent (C1S)	4.11
Signal transducer and activator of transcription 6, interleukin-4 induced (STAT6)	4.01
Lysyl oxidase-like 2 (LOXL2)	3.95
Myoferlin (MYOF)	3.64
Vimentin (VIM)	3.64
Endosialin (CD248)	3.42
Actin, gamma 2, smooth muscle, enteric (ACTG2)	3.36
**Epithelial membrane protein 1 (EMP1)**	3.35
Ubiquitin D (UBD)	3.11
Guanylate binding protein 3 (GBP3)	3.09

Table S2. Volume of tumors formed at the primary site of injection of LNCaP cells.

Cell type	n	Volume (mm^3)
Control LNCaP	6	58.4 ± 21.8
FLAG-EMP1-LNCaP	11	64.3 ± 43.2

Data are expressed as mean ± S.E.M.
There is no statistical significance between the two cell types.

Table S3. Clinical characteristics of patients whose prostate cancer samples were analyzed in this study.

Patient No.	Gleason score	TNM classification	Nuclear grade	Area of cancer invasion						Sample acquisition
				Extraprostatic extension	Resection margin	Lymphatic vessels	Blood vessels	Seminal vesicles	Perineurium	
LGS-1	7	pT2a	II	-	-	-	-	-	-	Operation
LGS-2	7	pT3a	II	+	-	-	-	-	+	Operation
LGS-3	6	pT2a	II	-	-	-	-	-	+	Operation
LGS-4	7	pT2c	II	-	-	-	-	-	-	Operation
LGS-5	7	pT2c	II	-	-	-	-	-	+	Operation
LGS-6	6	ypT1a	II	-	-	-	-	-	-	Operation
HGS-1	9	pT2b	II	-	±	+	+	-	+	Operation
HGS-2	9	cT4	N/A	N/A	N/A	N/A	N/A	N/A	N/A	Biopsy
HGS-3	8	pT3a	II	+	+	+	-	-	+	Operation
HGS-4	9	pT2c	II	-	-	+	+	-	+	Operation
HGS-5	9	pT3a	II	+	-	+	+	-	+	Operation
HGS-6	9	pT2c	II	-	-	-	-	-	+	Operation

HGS-2: surgical opreration was not performed due to the advanced age.
N/A: not applicable.

Table S4. List of the primary antibodies used for western blotting.

Name	Source	Type	Company	Catalogue No.	Dilution ratio
DYDDDDK <FLAG-tag> (1E6)	Mouse	Monoclonal	Wako Pure Chemical Industries	018-22381	1:1,000
GAPDH (3H12)	Mouse	Monoclonal	Medical & Biological Laboratories	M171-3	1:1,000
GFP (mFX75)	Mouse	Monoclonal	Wako Pure Chemical Industries	012-22541	1:1,000
LDL receptor (EP1553Y)	Rabbit	Monoclonal	Abcam	ab52818	1:1,000
Copine-III	Rabbit	Polyclonal	GeneTex	GTX107896	1:1,000
EMP1	Rabbit	Polyclonal	Originally generated		1:500
Rac1 (102)	Mouse	Monoclonal	BD Bioscience	610650	1:500
Src	Rabbit	Polyclonal	Cell Signaling Technology	2108	1:1,000
P-Src [Tyr416]	Rabbit	Polyclonal	Cell Signaling Technology	2101	1:500
Vav2 (C64H2)	Rabbit	Monoclonal	Cell Signaling Technology	2848	1:1,000
β-Actin (C4)	Mouse	Monoclonal	Santa Cruz Biotechnology	sc-47778	1:1,000
Phosphotyrosine (4G10)	Mouse	Monoclonal	GeneTex	GTX14167	1:500

Clone name is indicated in ( ).

Figure S1

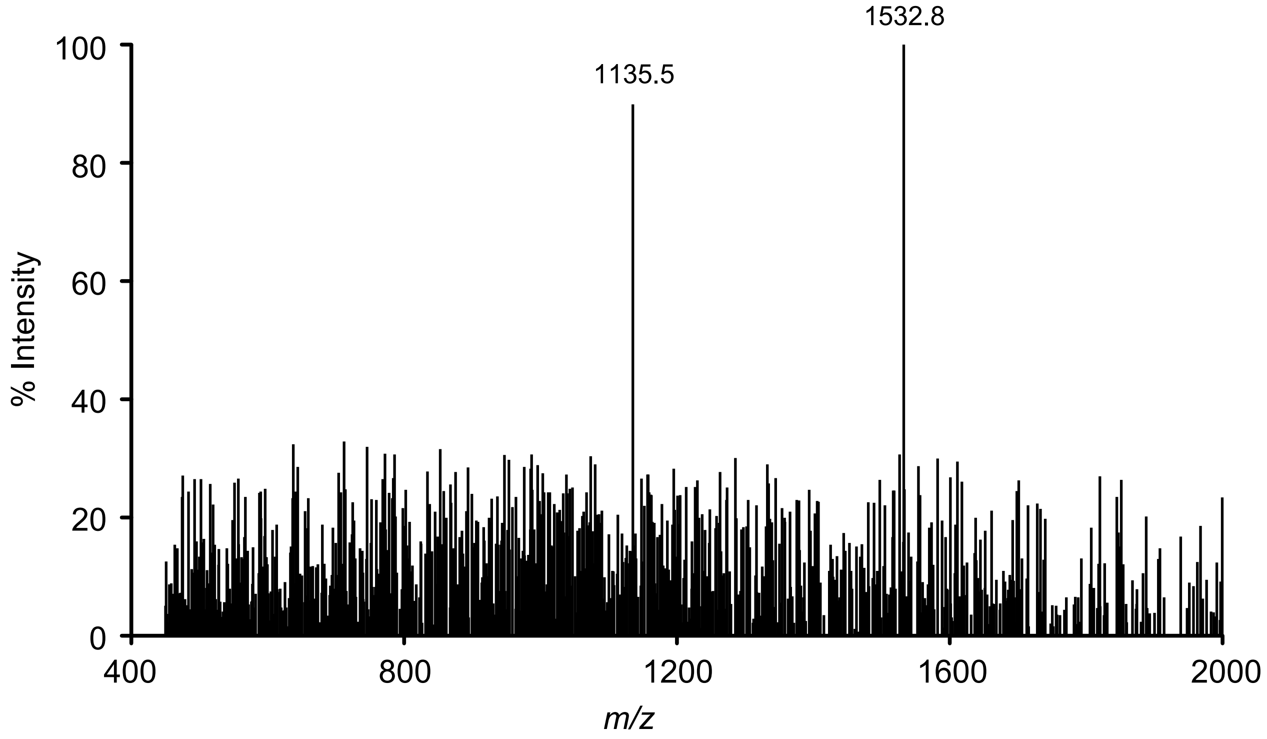

	Molecular mass	Sequence	Source	Accession number	Position
Peptide 1	1135.5	SDPYLEFHK	Copine-III	AAH07017	159-167
Peptide 2	1532.8	GSITISAEEIKDNR	Copine-III	AAH07017	126-139

Figure S2

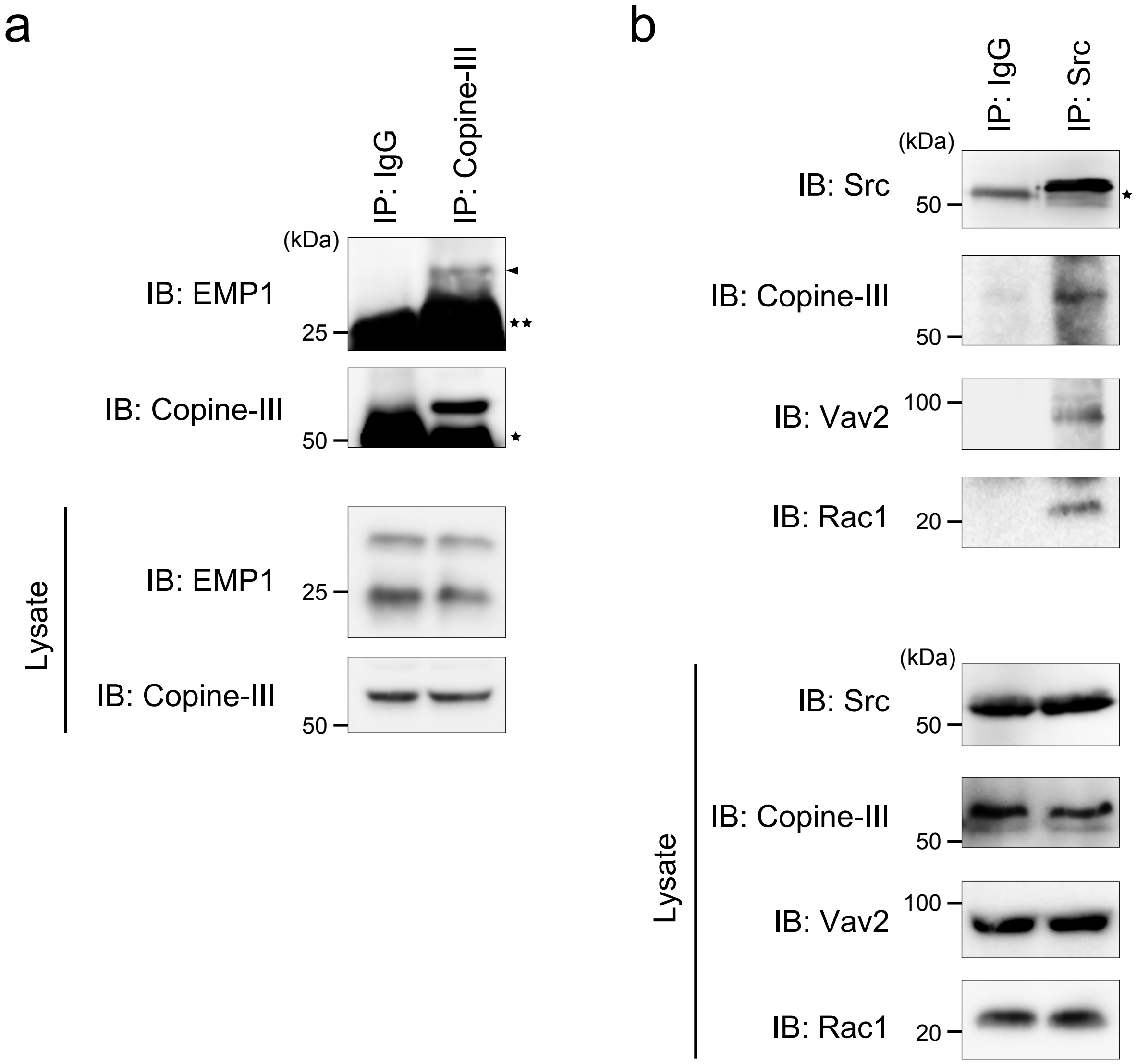
a
IP: IgG
IP: Copine-III
(kDa)
IB: EMP1
25
IB: Copine-III
50
Lysate
IB: EMP1
25
IB: Copine-III
50
b
IP: IgG
IP: Src
(kDa)
IB: Src
50
IB: Copine-III
50
IB: Vav2
100
IB: Rac1
20
(kDa)
Lysate
IB: Src
50
IB: Copine-III
50
IB: Vav2
100
IB: Rac1
20

Figure S3

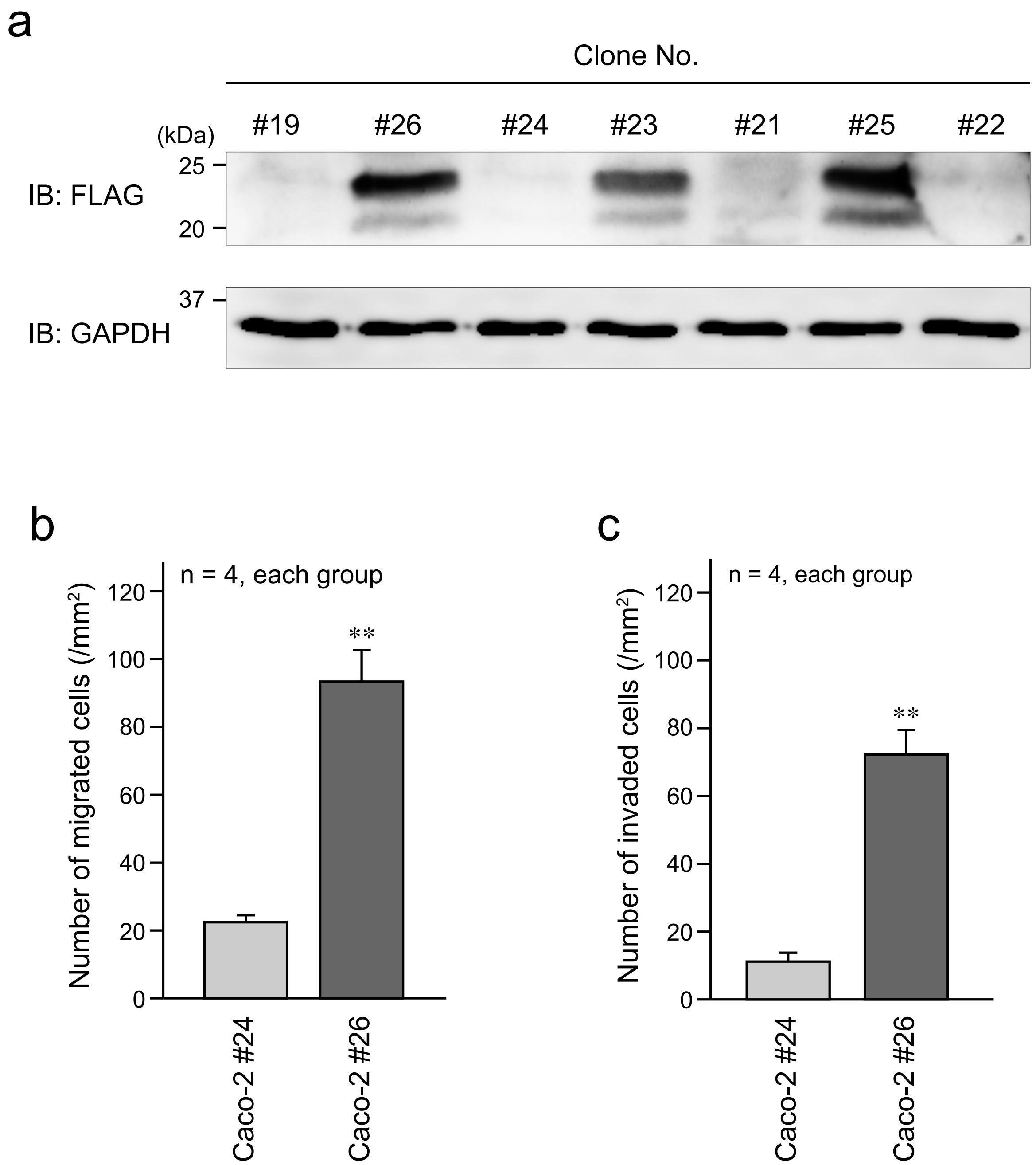
a
Clone No.
#19
#26
#24
#23
#21
#25
#22
(kDa)
25
20
37
IB: FLAG
IB: GAPDH
b
n = 4, each group
Number of migrated cells (/mm²)
120
100
80
60
40
20
0
**
Caco-2 #24
Caco-2 #26
c
n = 4, each group
Number of invaded cells (/mm²)
120
100
80
60
40
20
0
**
Caco-2 #24
Caco-2 #26
